# Genotype–environment interactions affecting preflowering physiological and morphological traits of *Brassica rapa* grown in two watering regimes

**DOI:** 10.1093/jxb/ert434

**Published:** 2014-01-27

**Authors:** Mohamed El-Soda, Martin P. Boer, Hedayat Bagheri, Corrie J. Hanhart, Maarten Koornneef, Mark G. M. Aarts

**Affiliations:** ^1^Laboratory of Genetics, Wageningen University, Droevendaalsesteeg 1, 6708 PB Wageningen, The Netherlands; ^2^Department of Genetics, Faculty of Agriculture, Cairo University, Egypt; ^3^Biometris–Applied Statistics, Department of Plant Science, Wageningen University, Wageningen, The Netherlands; ^4^Bu-Ali Sina University, Shahid Fahmideh, Hamedan, Iran; ^5^Max Planck Institute for Plant Breeding Research, Cologne, Germany

**Keywords:** Antagonistic fitness effect, *Brassica rapa*, drought, genotype–environment interaction, plasticity, root/shoot ratio, stomatal conductance.

## Abstract

Plant growth and productivity are greatly affected by drought, which is likely to become more threatening with the predicted global temperature increase. Understanding the genetic architecture of complex quantitative traits and their interaction with water availability may lead to improved crop adaptation to a wide range of environments. Here, the genetic basis of 20 physiological and morphological traits is explored by describing plant performance and growth in a *Brassica rapa* recombinant inbred line (RIL) population grown on a sandy substrate supplemented with nutrient solution, under control and drought conditions. Altogether, 54 quantitative trait loci (QTL) were identified, of which many colocated in 11 QTL clusters. Seventeen QTL showed significant QTL–environment interaction (Q×E), indicating genetic variation for phenotypic plasticity. Of the measured traits, only hypocotyl length did not show significant genotype–environment interaction (G×E) in both environments in all experiments. Correlation analysis showed that, in the control environment, stomatal conductance was positively correlated with total leaf dry weight (DW) and aboveground DW, whereas in the drought environment, stomatal conductance showed a significant negative correlation with total leaf DW and aboveground DW. This correlation was explained by antagonistic fitness effects in the drought environment, controlled by a QTL cluster on chromosome A7. These results demonstrate that Q×E is an important component of the genetic variance and can play a great role in improving drought tolerance in future breeding programmes.

## Introduction

Plant growth is greatly affected by environmental abiotic stresses, of which drought is the most common factor impeding crop productivity. Drought is likely to become more threatening with the predicted global temperature increase (Smith and De Smet, 2012). Three categories of plant adaptive strategies to drought have been recognized: drought escape by early flowering, drought tolerance via increasing water use efficiency and drought avoidance via reduced transpiration and increasing water uptake (Levitt, 1972).

Evaluating those responses in many genotypes in several environments may show phenotypic plasticity, which is defined as the ability of an individual organism to alter its physiology/morphology in response to changes in environmental conditions (Schlichting, 1986). When this plasticity differs between genotypes (i.e. when there is genetic variation for it), it is classified as genotype–environment interaction (G×E) (Via and Lande, 1985). Understanding G×E better will provide a solid foundation for genetic improvement of stable crop productivity and will help to identify superior and stable alleles/genotypes across different environments (Zhang *et al.*, 2010). The genetic basis of the observed G×E can be identified by genetically dissecting plant physiological and morphological responses to environments via quantitative trait loci (QTL). This specifies the genetic component of G×E and is expressed as QTL–environment interaction (Q×E) (Malosetti *et al.*, 2004; Boer *et al.*, 2007; Tardieu, 2013). Different QTL effects can occur if the allele underlying the QTL is strongly expressed in one environment but weakly in another, or if the allele has opposite effects on the same trait in different environments (Mackay, 2001; Sukhwinder *et al.*, 2012). A QTL for which one allele has opposite (pleiotropic) effects on the phenotype in two different environments can lead to fitness trade offs, elevating fitness in one environment but depressing it in the other environment. Trade offs can be maintained in nature (e.g. by antagonistic pleiotropy), when alleles at a locus underlying a fitness component show clear home-site advantages (Rose, 1982; Anderson *et al.*, 2013). Therefore, considering such antagonistic fitness effects is crucial while selecting for desirable QTL during marker-assisted breeding programmes.

To facilitate improving marker-assisted breeding programmes, a model crop plant is required. The *Brassica* genus has the smallest genome size, the complete genome sequence of *Brassica rapa* (Wang *et al.*, 2011), close relationships with the plant model species *Arabidopsis thaliana* and genome analysis tools, provided in the *Brassica* database (BRAD) (Cheng *et al.*, 2011), so *B. rapa* is a useful dicot model crop for genetic and comparative studies.

The present study focused on drought avoidance, which enables plants to maintain a high fitness level in drought conditions. Therefore, Q×E on growth-related traits were investigated in a *B. rapa* recombinant inbred line (RIL) population grown on a sandy substrate under control and drought environments. This work identified several QTL for main effects and Q×E and found an antagonistic fitness effect for a stomatal conductance/shoot biomass QTL, with the same allele reducing stomatal conductance under drought and increasing it under normal watering conditions, while contributing to higher shoot biomass in both environments.

## Materials and methods

### Plant material and experimental setup

The RIL population (F7) used here was previously developed by this study group from a cross between a Yellow Sarson (R-o-18) (♂) and a Caixin type (L58) (♀) and genotyped with 270 markers (Bagheri *et al.*, 2012). The RIL population was screened three times under control (continuous watering for 3 weeks) and drought (normal watering for 1 week, then plants were left to dry out) environments. In all screens, plants were grown in 13-cm-deep square black plastic pots. Each pot was filled with 1.5kg dried river sand and all pots were watered until saturation with 1100ml nutrient solution (1, 1.1, 5.9 mmol l^–1^, N, P, and K respectively). The same nutrient solution was used for watering plants every 2 days. Two seeds were sown per pot and 4 days after germination, seedlings were thinned to one per pot. Seven days after germination, watering was withheld as drought treatment, while the control treatment was continuously watered.

Initially, a pilot experiment was performed using 30 randomly selected RILs and both parental lines, with three replications per genotype per environment, to test if the drought treatment would reveal significant differences between RILs and between the two environments regarding total leaf fresh and dry weight. Subsequently, a full RIL screening experiment was performed in which 140 RILs and both parents were phenotyped for the 20 studied traits under both environments with three replications per RIL and six replications per parental line per treatment. Finally a QTL reproducibility experiment was performed to confirm the different phenotypes for contrasting alleles at four identified QTL by screening 27 RILs selected for their discriminating genotypes, with three replicates per RIL per environment.

All experiments were carried out under controlled greenhouse conditions under a16/8 light/dark cycle (22.3/20.3°C, mean relative humidity 77.8/81.3%). The experimental setup involved a complete randomized block design with one plant per RIL and two replicates for each parent per block.

### Plant phenotyping

In the full RIL screening and QTL reproducibility experiments, 20 traits were analysed under control and drought environments. These traits were chosen as the ones describing as best as possible the different aspects of plant performance. Directly before harvesting, when less than 5% of plants had visible flower primordia, the number of leaves was counted. Chlorophyll content was measured (only in the full RIL screening experiment) using a SPAD-502 chlorophyll meter (Minolta, Japan). For this measurement the average of three leaves per plant per replication per treatment was taken. Leaf stomatal conductance was measured using a leaf porometer (Decagon Devices, USA) for one fully expanded leaf per plant per replication (either the 3rd or 4th leaf). Thereafter, total leaf fresh weight (LFW) and dry weight (LDW) and the dry weight of the 3rd and 4th (i.e. fully expanded) leaves (3,4DW) was measured. Dry weights were determined after drying plant materials at 65°C for 4–5 days until weight constancy.

Leaf area (LA) of the 3rd and 4th leaves was measured using a Licor LI-3100 (Licor, Lincoln, NE, USA), and subsequently their combined specific leaf area (SLA) was calculated as LA divided by 3,4DW, as well as the dry weight ratio between 3,4DW and LDW. Hypocotyl length was measured using a ruler, and hypocotyl DW (HDW) was determined. The shoot DW (SDW) was calculated as the sum of LDW and HDW.

Subsequently, root systems were washed carefully to remove adhering sand, placed in a plastic tray filled with water, spread and scanned with a flatbed scanner. From this, the total root system length (RL), root volume (RV), and root diameter (RD) were measured using WinRhizo (Regent Instruments, Quebec, Canada). This was used to calculate the RL-to-SDW ratio (RL/SDW), which illustrates the aboveground matter that is supported by a given RL.

Thereafter, roots were dried to measure root DW (RDW) and to calculate the root-to-shoot DW ratio (R/S). Similarly, to indicate the relative investment in shoots or roots, the shoot-to-total plant (shoot + root) DW ratio was calculated (S/SR), for which total plant DW was calculated as the sum of SDW and RDW. Finally, the leaf water content (LWC) was calculated as (LFW – LDW) / LDW.

### Statistical and quantitative trait loci analysis

Statistical analysis was performed on raw data of each experiment using GenStat for Windows 15th edition (VSN International, Hemel Hempstead, UK). Analysis of variance (ANOVA) was used to test the significance difference between treatments, lines, and interaction (G×E). Heritability was estimated as implemented in GenStat. In the linear mixed model, genotypes were fitted as random and blocks as fixed. The generalized heritability measure used, as described by Cullis *et al.* (2006), and in a more general context by Welham *et al.* (2010), is given by:

$$h^{2}=\text{1}-\;\frac{\text{mean}\left(\text{pev}\left(g_{i}\right)\right)}{\sigma_{g}^{2}}$$

where the set of predicted genotype means (Best Linear Unbiased Predictors) are *g*_1_ …* g*_*N*_ with prediction error variance pev(*g*_*i*_) and estimated genetics variance component $\sigma_{g}^{2}$. Pearson correlations were calculated using GenStat.

Data from the 20 traits analysed in the full RIL screening experiment were used for QTL mapping using a multienvironment analysis (MEA) approach, which accounts for G×E, as implemented in the QTL library in GenStat. A step size of 10 cM, a minimum cofactor proximity of 50 cM, a minimum separation of selected QTL of 30 cM, and a threshold of –log10P = 2.8 were used for QTL analysis. Following the mixed-model approach described by (Malosetti *et al.*, 2004; Boer *et al.*, 2007), first the whole genome was scanned using simple interval mapping and then, based on that, cofactors were selected for two rounds of composite interval mapping. Thereafter, a final QTL model was selected using backward selection on the selected cofactors, where it estimated the allelic effect of each of QTL in each environment, the effect of Q×E, and the explained phenotypic variance of each QTL per environment. In addition to determining phenotypic plasticity as Q×E, a second method to determine plasticity QTL was used as described by (Tétard-Jones *et al.*, 2011), by QTL mapping the difference in the mean phenotypic values per line between treatments.

### Confirming reproducibility of four QTL clusters

To confirm the reproducibility of the major QTL detected in the full RIL screening experiment, this work selected four QTL clusters: on chromosome 3 at 38–42 cM, on chromosome 7 at 30–40 cM, on chromosome 8 at 85–95 cM, and on chromosome 9 at 70–84 cM. The whole population was genetically classified into 16 groups based on all possible allelic combinations at the four selected QTL. Thereafter, for every tested QTL, phenotypic data of RILs with contrasting genotypes for one QTL, but similar genotypes for the other QTL, were compared. For example, to test for the QTL on chromosome 3, ANNN RILs were compared with BNNN RILs in paired groups, so AAAA with BAAA, ABAA with BBAA, ABBA with BBBA, etc. The 27 RILs with the highest and lowest average values at each tested QTL were selected and grown as described. For all measured traits, a correlation analysis between traits measured in the control environments and traits measured in the drought environments of the full RIL screening experiment and the QTL reproducibility experiments was used to test for a significantly similar response to the treatment as a confirmation of the level of reproducibility.

## Results

### Phenotyping the RIL population

**Fig. 1.**
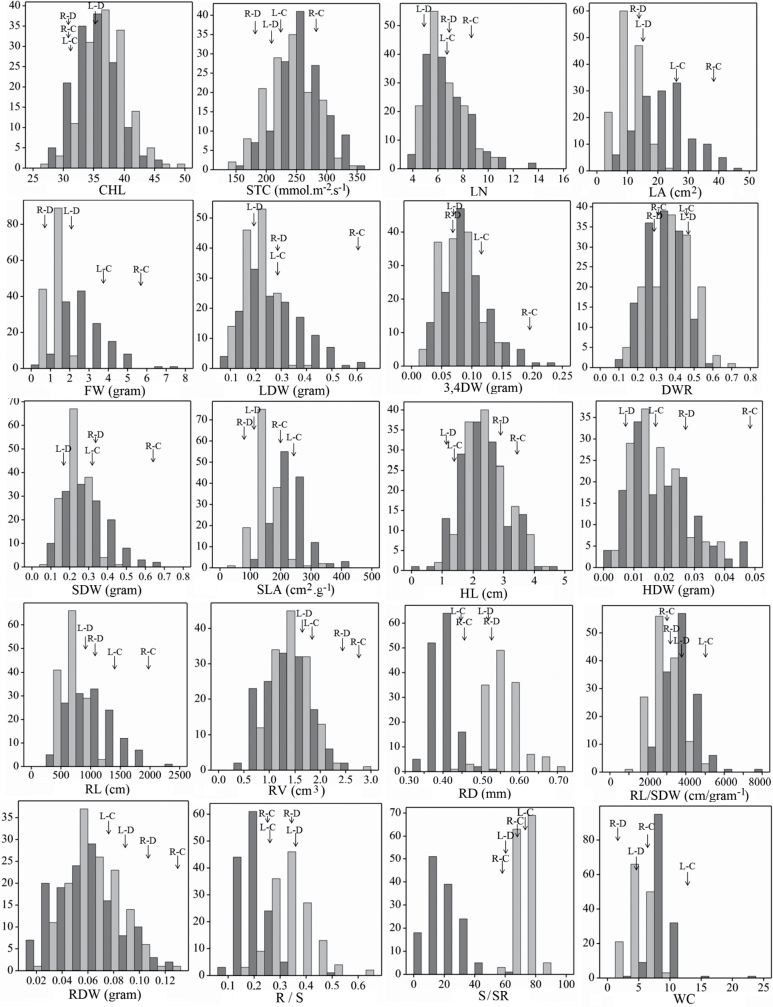
Frequency distributions of the non-normalized trait values for the L58 × R-o-18 recombinant inbred line population under control (C) (dark grey), and drought (D) (light grey) conditions. Vertical axes indicate the number of lines per trait value class, and horizontal axes indicate the different trait value classes. L, L58; R, R-o-18. Trait abbreviations are given with Table 1.

The results obtained from the pilot experiment (data not shown) indicated there was ample phenotypic variation for drought response, which justified phenotyping the whole RIL population. A total of 20 traits related to growth and performance of plants were analysed under control and drought environments. Fig. 1 shows the frequency distributions of the measured traits over the whole population. Transgression beyond both parental lines was observed for most of the traits except for root volume, RDW, HDW, SDW, S/SR, and LWC, where transgression was only in one direction.

**Table 1.** Parental line means for the analysed traits and performance of the phenotyped RIL population under control and drought conditionsFor all traits, three replicates were measured. Min and Max indicate the lowest and highest RIL values; mean and standard deviation (SD) is for all RILs; h^2^ indicates broad sense heritability. S, significant; NS, nonsignificant; –, not applicable (ANOVA and heritability cannot be calculated because averages and not replications were used). CHL, chlorophyll content; HDW, hypocotyl dry weight; HL, hypocotyl length; LA, leaf area; LDW, leaf dry weight; LFW, leaf fresh weight; LN, leaf number; LWC, leaf water content; RD, root diameter; RDW, root dry weight; RIL, recombinant inbred line; RL/SDW, root length-to-shoot dry weight ratio; RL, root system length; RV, root volume; R/S, root-to-shoot dry weight ratio; SDW, shoot dry weight; S/SR, shoot-to-plant dry weight ratio; SLA, specific leaf area; STC, stomatal conductance; DWR, 3,4DW vs. LDW ratio; 3,4DW, 3rd and 4th leaf dry weight.

Trait and treatment	Unit	Parental lines		RIL population				ANOVA 2nd experiment			h^2^	ANOVA 3rd experiment		
		L58	R-o-18	Min	Max	Mean	SD^b^	Treatment	RILs	G×E		Treatment	RILs	G×E
CHL with C		31.84	31.45	28.20	47.45	35.77	3.47	S	S	NS	0.59			
CHL with D		35.14	30.96	27.40	47.70	36.87	3.58				0.50			
STC with C	mmol m^–2^ s^–1^	237.87	266.12	173.95	364.25	265.50	37.59	S	S	S	0.31	S	NS	NS
STC with D		220.50	165.67	125.90	340.70	231.80	39.17				0.81			
LN with C	*n*	6.67	7.50	4.50	14.00	7.24	1.75	S	S	S	0.56	S	S	NS
LN with D		5.60	6.60	3.67	10.33	6.20	1.42				0.78			
LA with C	cm^2^	26.38	35.79	6.33	47.91	23.73	8.47	S	S	NS	0.26	S	S	S
LA with D		11.87	10.04	1.28	22.24	9.23	3.83				0.46			
LFW with C	g	3.68	5.11	0.772	7.458	2.994	1.136	S	NS	NS	0.44	S	S	S
LFW with D		1.21	0.77	0.268	2.046	0.979	0.324				0.36			
LDW with C	g	0.276	0.619	0.087	0.637	0.293	0.114	S	S	NS	0.31	S	S	S
LDW with D		0.196	0.275	0.060	0.370	0.193	0.057				0.37			
3,4DW with C	g	0.112	0.194	0.032	0.231	0.099	0.039	S	S	NS	0.48	S	S	S
3,4DW with D		0.082	0.081	0.017	0.150	0.069	0.029				0.57			
DWR with C		0.406	0.314	0.106	0.561	0.356	0.096	–	–	–	–	–	–	–
DWR with D		0.419	0.295	0.098	0.670	0.366	0.117				–			
SDW with C	g	0.288	0.671	0.092	0.665	0.315	0.122	–	–	–	–	–	–	–
SDW with D		0.205	0.300	0.074	0.408	0.210	0.062				–			
SLA with C	cm^2^ g^–1^	235.03	184.48	139.00	426.35	246.20	52.43	–	–	–	–	–	–	–
SLA with D		144.45	123.95	35.42	325.75	137.60	42.89				–			
HL with C	cm	1.60	3.40	0.500	4.550	2.439	0.738	S	S	NS	0.90	S	S	NS
HL with D		1.10	2.78	0.733	4.300	2.373	0.692				0.91			
HDW with C	g	0.012	0.052	0.003	0.048	0.021	0.011	S	S	NS	0.33	S	S	S
HDW with D		0.009	0.025	0.004	0.038	0.017	0.008				0.60			
RL with C	cm	1398.50	1970.45	304.40	2300.00	1078.00	388.60	S	S	S	0.48	S	S	S
RL with D		741.80	940.92	254.79	1249.64	616.60	183.40				0.48			
RV with C	cm^3^	1.71	2.98	0.442	2.696	1.381	0.455	NS	S	NS	0.38	NS	S	S
RV with D		1.52	2.21	0.697	2.990	1.379	0.361				0.46			
RD with C	mm	0.395	0.424	0.340	0.560	0.411	0.030	S	S	S	0.36	NS	S	S
RD with D		0.500	0.550	0.429	0.686	0.548	0.046				0.26			
RL/SDW with C	cm g^–1^	4849.20	2936.90	2088.00	7298.00	3545.00	797.40	–	–	–	–	–	–	–
RL/SDW with D		3616.80	3138.90	1502.00	5525.00	3049.00	750.30				–			
RDW with C	g	0.070	0.157	0.013	0.122	0.060	0.025	S	S	NS	0.29	S	S	S
RDW with D		0.080	0.100	0.020	0.124	0.063	0.020				0.47			
R/S with C		0.242	0.233	0.097	0.410	0.192	0.047	–	–		–	–	–	–
R/S with D		0.369	0.339	0.151	0.557	0.308	0.071				–			
S/SR with C		77.13	74.75	2.04	60.30	21.53	10.84	–	–	–	–	–	–	–
S/SR with D		69.94	68.45	55.32	93.97	70.62	4.66				–			
LWC with C		12.31	7.26	0.684	7.362	2.701	1.043	–	–	–	–	–	–	–
LWC with D		5.16	1.80	0.110	1.765	0.786	0.300				–			

The drought treatment decreased fresh weight, leaf number, leaf area, LDW, root length, and stomatal conductance and increased R/S (Fig. 1, Table 1). For stomatal conductance, the reduction in the L58 parent was minor and not significant, as was also the case for some of the RILs.

Correlation analysis of all measured traits in this experiment was performed to unveil the genetic and physiological relationships of the various traits (Table 2). The correlations may exist because of similar physiological mechanisms or pleiotropy; however, correlations can also be caused by genetic linkage of loci affecting different traits, which are not physiologically related or pleiotropic. For instance, the analysis showed that, in the control environment, chlorophyll content was positively correlated with root diameter, which is hard to envision being because of pleiotropy.

**Table 2.** Pearson correlations for the analysed traits of the L58 × R-o-18 RIL population under control (A) and drought (B) conditionsHighlighted results refer to significant correlations: dark grey, *P* < 0.01; light grey, *P* < 0.05. Trait abbreviations are given with Table 1.

A																					
CHL	1	-																			
STC	2	-0.017	-																		
LN	3	-0.075	0.115	-																	
LA	4	-0.027	0.114	-0.235	-																
LFW	5	-0.010	0.188	0.448	0.571	-															
LDW	6	0.079	0.207	0.441	0.538	0.939	-														
3,4DW	7	0.096	0.129	-0.125	0.851	0.668	0.737	-													
DWR	8	0.009	-0.172	-0.776	0.366	-0.410	-0.412	0.265	-												
SDW	9	0.072	0.207	0.443	0.539	0.943	0.998	0.735	-0.413	-											
SLA	10	-0.260	-0.025	-0.161	0.168	-0.224	-0.404	-0.333	0.130	-0.399	-										
HL	11	-0.128	0.116	-0.092	0.116	0.150	0.102	0.135	0.015	0.147	-0.039	-									
HDW	12	-0.023	0.139	0.329	0.387	0.696	0.671	0.493	-0.296	0.719	-0.228	0.562	-								
RL	13	0.037	0.087	0.471	0.457	0.800	0.831	0.629	-0.339	0.830	-0.352	-0.028	0.569	-							
RV	14	0.100	0.106	0.460	0.464	0.770	0.797	0.619	-0.311	0.797	-0.338	-0.021	0.558	0.929	-						
RD	15	0.161	-0.037	-0.169	-0.181	-0.349	-0.355	-0.250	0.160	-0.357	0.134	-0.033	-0.272	-0.475	-0.190	-					
RL/SDW	16	-0.042	-0.215	0.018	-0.152	-0.259	-0.316	-0.205	0.193	-0.321	0.141	-0.312	-0.276	0.218	0.165	-0.175	-				
RDW	17	0.087	0.105	0.459	0.445	0.778	0.810	0.627	-0.321	0.818	-0.380	0.021	0.649	0.891	0.929	-0.245	0.057	-			
R/S	18	0.068	-0.086	0.140	-0.061	-0.053	-0.102	-0.035	0.082	-0.084	-0.008	-0.088	0.123	0.264	0.358	0.096	0.660	0.441	-		
S/SR	19	0.084	0.161	0.410	0.483	0.830	0.905	0.684	-0.370	0.904	-0.393	0.041	0.623	0.820	0.790	-0.324	-0.190	0.819	0.057	-	
WC	20	-0.224	-0.112	-0.078	-0.044	-0.039	-0.336	-0.289	0.114	-0.320	0.559	0.106	-0.066	-0.227	-0.228	0.086	0.299	-0.238	0.247	-0.327	-
		1	2	3	4	5	6	7	8	9	10	11	12	13	14	15	16	17	18	19	20
B																					
CHL	1	-																			
STC	2	0.029	-																		
LN	3	0.000	-0.075	-																	
LA	4	-0.035	-0.107	-0.320	-																
LFW	5	0.013	-0.080	0.131	0.571	-															
LDW	6	-0.047	-0.189	0.404	0.473	0.494	-														
3,4DW	7	-0.098	-0.213	-0.226	0.773	0.455	0.637	-													
DWR	8	-0.051	-0.062	-0.685	0.557	0.121	-0.141	0.653	-												
SDW	9	-0.066	-0.187	0.405	0.463	0.473	0.995	0.618	-0.160	-											
SLA	10	0.072	0.141	-0.122	0.305	0.123	-0.119	-0.283	-0.218	-0.103	-										
HL	11	-0.141	0.156	-0.090	-0.027	0.041	0.022	-0.013	-0.041	0.083	-0.007	-									
HDW	12	-0.174	-0.107	0.278	0.248	0.175	0.635	0.282	-0.236	0.711	0.040	0.481	-								
RL	13	0.005	-0.169	0.198	0.404	0.130	0.646	0.429	-0.053	0.648	0.032	-0.156	0.453	-							
RV	14	-0.010	-0.280	0.288	0.392	0.273	0.664	0.460	-0.034	0.662	-0.037	-0.099	0.429	0.828	-						
RD	15	-0.042	-0.093	0.063	-0.261	0.061	-0.204	-0.180	-0.061	-0.214	-0.146	0.116	-0.219	-0.556	-0.084	-					
RL/SDW	16	0.067	0.045	-0.289	-0.103	-0.429	-0.452	-0.226	0.175	-0.452	0.147	-0.262	-0.303	0.340	0.116	-0.434	-				
RDW	17	-0.047	-0.181	0.325	0.370	0.224	0.708	0.423	-0.116	0.725	-0.017	-0.029	0.607	0.813	0.845	-0.240	0.029	-			
R/S	18	-0.025	0.001	-0.110	-0.133	-0.326	-0.317	-0.211	0.063	-0.286	0.060	-0.037	0.019	0.217	0.235	-0.057	0.622	0.404	-		
S/SR	19	0.080	0.010	0.102	0.122	0.328	0.256	0.212	-0.002	0.196	-0.088	-0.190	-0.280	-0.227	-0.227	0.094	-0.530	-0.420	-0.940	-	
WC	20	0.043	0.126	-0.314	0.085	0.449	-0.505	-0.158	0.302	-0.517	0.240	0.047	-0.432	-0.521	-0.389	0.279	0.055	-0.493	0.010	0.043	-
		1	2	3	4	5	6	7	8	9	10	11	12	13	14	15	16	17	18	19	20

The correlation observed between root length and S/SR was positive in the control environment (longer roots contributing to relatively more shoots) but negative in the drought environment, indicating a proportionally higher investment in roots. Under both environments, LWC was negatively correlated with SDW, root length, root volume and RDW, while it was positively correlated with LFW and negatively correlated with stomatal conductance under drought conditions. Stomatal conductance was negatively correlated with LDW in the drought environment, but positively correlated under control conditions. In general, plants with longer root systems had higher plant DW.

As expected, all traits measured in control and drought environments showed a positive correlation, except for LWC.

### Mapping QTL with main effects and Q×E

**Fig. 2.**
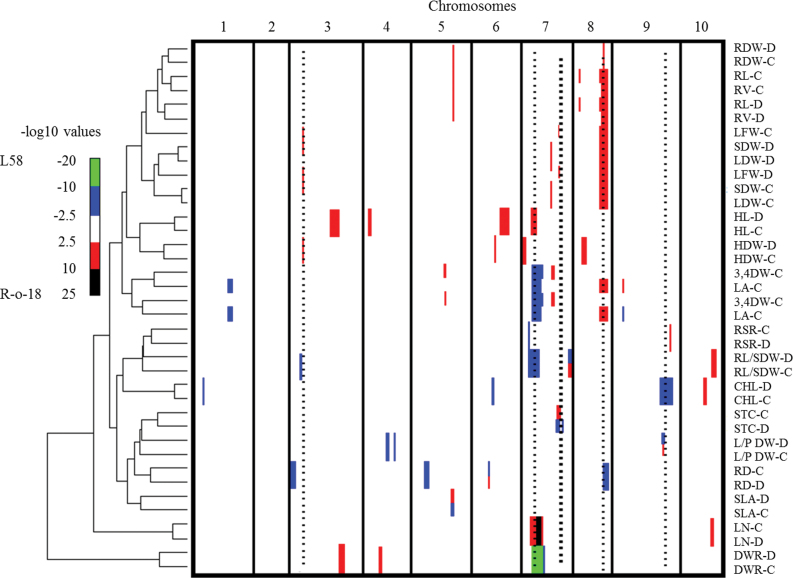
A clustered heat map showing the –log10P profiles of the measured traits. Columns indicate the 10 chromosomes in centiMorgans, ascending from left to right; rows indicate individual trait –log10P profiles. A colour scale is used to indicate the QTL significance corresponding to the –log10P score: red and black represent a positive effect on the trait value from the R-o-18 allele; blue and green represent a positive effect on the trait value from the L58 allele. Bar width indicates the significance interval of the QTL. Hierarchical clustering, shown on the left, reflects the correlation between traits based on the QTL profiles. Right dotted line of heavier weight in A7 indicates the QTL with an antagonistic fitness effect; the other five dotted lines refer to QTLs confirmed in the reproducibility experiment. C and D refer to control and drought environments, respectively. Trait abbreviations are given with Table 1.

In total 54 QTL were mapped for the traits analysed under control and drought environments (Table 3, Fig. 2). Six QTL—*STC1*, *LA4*, *SLA1*, *RD3*, *RL/SDW3*, and *S/SR2*—had opposite allelic effects when comparing both environments. The phenotypic effects of three QTL—*LA1*, *SLA1*, and *S/SR2*—were 9-, 101-, and 15-times higher, respectively, in one environment than the other (Table 3). *SLA1* colocated with *3,4DW1*, with the alleles increasing the trait values in the control environment from L58 and R-o-18, respectively. Four QTL were mapped for chlorophyll content, of which *CHL1*, *CHL2*, and *CHL3* showed the highest effect from the L58 allele, while for *CHL4* the R-o-18 allele had the highest effect in both environments. Hypocotyl length was mapped to four loci, with the R-o-18 alleles contributing most to increased hypocotyl length. In total, 11 QTL clusters were observed, of which seven comprised at least three colocating QTL (Table 3, Fig. 2).

**Table 3.** QTL detected in the L58 × R-o-18 RIL population for the traits described in Table 1, using the multienvironment analysis approachPer trait, QTL are numbered according to chromosome. *R* ^*2*^ is the percentage of total phenotypic variance explained by each QTL. Effects with positive values represent a positive contribution of the R-o-18 allele to the trait value and those with negative values represent a positive contribution of the L58 allele to the trait value. Highlighted results show significant Q×E effects. Ratio refers to the ratios between the effects of each QTL in both environments. Trait abbreviations are given with Table 1.

Trait	QTL				Control		Drought		Ratio
	Name	Linkage group	Position of highest peak (cM)	–log10P	Effect	R^2^	Effect	R^2^	
CHL	*CHL1*	A1	24.23	4.2	–0.855	5.7	–0.855	5.4	
	*CHL2*	A6	59.11	4.5	–0.885	5.9	–0.885	5.6	
	*CHL3*	A9	77.21	8.9	–1.304	14.2	–1.304	13.4	
	*CHL4*	A10	56.32	5.9	1.011	10.4	1.011	9.7	
STC	*STC1*	A7	96.75	3.0	6.395	1.9	–9.754	7.9	–1.5
LN	*LN1*	A7	40.75	11.1	0.799	15.6	11.103	23.5	
	*LN2*	A10	62.99	4.6	0.426	5.4	4.650	8.1	
LA	*LA1*	A1	70.54	6.0	–3.343	15.6	–0.358	0.9	9.3
	*LA2*	A7	32.05	4.8	–1.251	2.2	–1.251	10.7	
	*LA3*	A8	85.20	5.2	1.321	2.4	1.321	11.9	
	*LA4*	A9	24.28	3.4	1.865	4.8	–0.394	1.1	–4.7
LFW	*LFW1*	A3	42.66	3.2	0.313	7.6	0.062	3.7	5.0
	*LFW2*	A7	105.32	3.6	0.092	0.7	0.092	8.1	
	*LFW3*	A8	85.20	6.1	0.120	1.1	0.120	13.7	
LDW	*LDW1*	A3	42.66	3.5	0.036	9.9	0.010	3.0	3.7
	*LDW2*	A7	100.81	3.9	0.016	2.1	0.016	8.4	
	*LDW3*	A8	91.33	7.3	0.023	4.0	0.023	16.5	
3,4DW	*3,4DW1*	A5	69.71	2.8	0.011	8.7	0.003	1.3	3.4
	*3,4DW2*	A7	34.89	7.4	–0.011	8.5	–0.011	14.9	
	*3,4DW3*	A7	100.81	3.0	0.007	3.1	0.007	5.5	
DWR	*DWR1*	A3	120.61	3.9	0.024	6.1	0.024	4.2	
	*DWR2*	A4	75.90	2.9	0.020	4.4	0.020	3.0	
	*DWR3*	A7	40.75	18.3	–0.054	31.1	–0.076	42.8	1.4
SDW	*SDW1*	A3	42.66	3.2	0.038	9.7	0.011	2.9	3.6
	*SDW2*	A7	83.27	3.0	0.016	1.8	0.016	7.0	
	*SDW3*	A8	91.33	7.0	0.025	4.2	0.025	16.3	
SLA	*SLA1*	A5	60.82	4.6	–20.3	15.0	0.201	0.0	–101.4
HL	*HL1*	A3	94.58	6.6	0.245	11.0	0.245	12.5	
	*HL2*	A4	54.79	3.7	0.176	5.7	0.176	6.5	
	*HL3*	A6	101.21	6.6	0.265	12.9	0.265	14.7	
	*HL4*	A7	18.39	4.7	0.200	7.3	0.200	8.3	
HDW	*HDW1*	A3	38.29	4.9	0.003	4.9	0.003	8.8	
	*HDW2*	A6	62.85	4.0	0.002	3.8	0.002	6.8	
	*HDW3*	A7	3.99	6.8	0.004	14.0	0.002	5.9	2.0
	*HDW4*	A8	33.95	4.1	0.003	6.3	0.003	11.3	
RL	*RL1*	A5	69.71	4.1	126.3	10.6	30.215	2.7	4.2
	*RL2*	A8	21.23	3.3	120.1	9.6	32.583	3.2	3.7
	*RL3*	A8	86.57	5.0	62.1	2.6	62.124	11.5	
RV	*RV1*	A5	69.71	3.2	0.141	9.6	0.056	2.4	2.5
	*RV2*	A8	86.57	5.7	0.124	7.4	0.124	11.8	
RD	*RD1*	A3	5.92	5.6	–0.010	10.8	–0.010	4.6	
	*RD2*	A5	35.17	4.8	–0.008	7.8	–0.008	3.3	
	*RD3*	A6	48.53	2.6	–0.005	2.8	0.010	5.0	–2.0
	*RD4*	A8	95.50	4.3	–0.007	6.0	–0.007	2.6	
RL/SDW	*RL/SDW1*	A3	21.88	2.3	–133.0	2.8	–133.0	3.1	
	*RL/SDW2*	A7	18.39	6.2	–236.8	8.8	–236.8	10.0	
	*RL/SDW3*	A7	125.27	2.7	160.8	4.1	–142.8	3.6	–1.1
	*RL/SDW4*	A10	62.99	4.0	182.5	5.2	182.5	5.9	
RDW	*RDW1*	A5	69.71	3.1	0.005	4.4	0.005	6.7	
	*RDW2*	A8	86.57	4.9	0.006	6.7	0.006	10.3	
R/S	*R/S1*	A7	18.39	3.6	–0.014	8.2	–0.014	3.7	
	*R/S2*	A9	84.14	3.0	0.004	0.7	–0.021	8.5	5.1
S/SR	*S/SR1*	A4	90.12	3.2	–0.010	6.3	–0.010	4.7	
	*S/SR2*	A9	69.95	3.4	0.001	0.0	–0.013	8.2	–14.9

### Stomatal conductance QTL (*STC1*) and fitness trade offs in the drought environment

The correlation analysis showed that, in the control environment, stomatal conductance was positively correlated with LDW and SDW. On the other hand, in the drought environment, stomatal conductance showed a significant negative correlation with LDW and SDW. These correlations were associated with altering the trait-value-enhancing allele for *STC1* from R-o-18 in the control environment to L58 in the drought environment. The trait-value-enhancing alleles for the QTL colocating with *STC1* (*LFW2*, *LDW2*, *3*,*4DW2*) were R-o-18 in both environments (Table 3, Fig. 2). This means that the R-o-18 alleles for these loci were enhancing fitness both under control and drought conditions, although having contrasting phenotypic effects on stomatal conductance when comparing both conditions.

### Mapping QTL underlying plasticity

Seventeen of the mapped QTL showed a significant Q×E effect (Table 3) indicating the loci contributing to phenotypic plasticity between both environments. In addition to the GenStat method to determine these plasticity loci, an alternative method to describe QTL that are affected by the environments was suggested by Tétard-Jones *et al.* (2011). This uses the differences between the trait-value averages of the lines in the two environments to determine QTL. Using this procedure, 15 plasticity QTL were mapped (Table 4), with nine of them colocating with previously mapped QTL, six of which were found to show Q×E (Table 3). Thus, this analysis detected six new plasticity QTL, which did not exceed the statistical significance levels with the GenStat method.

**Table 4.** QTL mapped for phenotypic plasticity in the L58 x R-o-18 RIL populationPlasticity was calculated as described by Tétard-Jones *et al.* (2011), as the difference in the mean phenotype between different treatments per trait. QTLs are numbered according to chromosome. *R* ^*2*^ is the percentage of total plastic variance explained by each QTL. Effects with positive values represent a positive contribution of the R-o-18 allele to the trait value and those with negative values represent a positive contribution of the L58 allele to the trait value. Highlighted QTL were mapped before using the multienvironment analysis approach (Table 3). Trait abbreviations are given with Table 1.

Trait	QTL name	Locus	Chromosome	Position (cM)	Effect	–log10P	*R* ^2^
STC	*STC1*	903607|9917837	7	96.8	17.22	3.8	11.0
LA	*LA1*	E3835M11	1	69.0	–2.69	4.6	13.3
LDW	*LDW4*	E3850M9	5	69.7	0.03	3.2	9.2
3,4DW	*3,4DW3*	Ra2A01-A7	7	83.3	–0.01	2.9	8.3
SDW	*SDW4*	E3850M9	5	69.7	0.03	3.0	8.8
SLA	*SLA2*	E3749M6	1	94.1	17.26	3.0	7.4
	*SLA1*	BrID101239-A5	5	65.7	–22.36	4.0	12.4
HL	*HL5*	902225|9924661	8	95.5	–0.08	3.0	8.0
RL	*RL4*	E3732M5	1	92.1	–101.68	3.3	7.9
	*RL1*	E3850M9	5	69.7	96.41	2.9	7.1
	*RL3*	E3416M22	8	91.3	112.99	4.0	9.8
RV	*RV3*	E3732M5	1	92.1	–0.12	3.0	8.1
RD	*RD3*	899015|9918455	6	43.5	–0.02	3.6	9.7
RL/SDW	*RL/SDW3*	C7P119	7	119.0	304.08	3.4	11.8
R/S	*R/S2*	BrID10177-A9	9	68.3	–0.02	2.7	7.0

### Reproducibility

From the 11 QTL clusters that were mapped, four were selected to be tested for reproducibility in a subsequent experiment. The first cluster mapped to A3, including the *RD1* and *RL/SDW1* QTL for both positively correlated traits, with trait-value-enhancing effects from the L58 alleles. Moreover, *LFW1*, *LDW1*, and *SDW1*, all contributing to shoot biomass, were mapped to the same cluster, with positive alleles coming from R-o-18. The second cluster was mapped to A7—composed of *LN1*, *LA2*, *3,4DW2*, *DWR3*, *R/S1*, and *RL/SDW2*—all with a positive contribution of the R-o-18 allele except for *LN1*. This is in line with the negative correlation of leaf number with the other traits. The third cluster, on A8, included eight colocating QTL—*LFW3*, *LDW3*, *SDW3*, *LA3*, *RL3*, *RVl2*, *RD4*,and *RDW2*—of which the *RD4* L58 allele increased the trait value, while for the other QTL, the R-o-18 allele increased the trait value, in line with the negative correlation of RD with the other traits. The fourth cluster included three QTL—*CHL3*, *R/S2*, and *S/SR2*—mapping to A9. The S/SR ratio showed a negative correlation between control and drought environments and therefore the trait-value-enhancing effect of *S/SR2* in the drought environment came from the L58 allele, whereas in the control environment it came from the R-o-18 allele.

In total, 27 lines were selected from the RIL population to properly represent the 16 possible genotypes for all allelic combinations for the four selected QTL clusters. These lines were regrown under similar conditions and rephenotyped (Supplementary Table 1, available at *JXB* online). A correlation analysis (Table 5) between traits measured in the two control environments and between traits measured in the two drought environments of the full RIL screening and QTL reproducibility experiments showed that all traits were positively correlated in at least one environment, but often both, except for fresh weight, LWC, and root diameter. This indicates that the phenotyping was robust and the detected QTL clusters are reproducible, making them attractive candidates for further gene cloning experiments.

**Table 5.** Correlation analysis between the control conditions and the drought conditions of the full RIL screening experiment and the reproducibility experimentHighlighted results refer to significant correlations: dark grey, *P* < 0.01; light grey, *P* < 0.05. Trait abbreviations are given with Table 1.

Trait	Control	Drought
STC	0.260	0.494
LN	0.852	0.758
LA	0.504	0.220
LFW	0.122	–0.027
LDW	0.270	0.258
3,4DW	0.474	0.239
DWR	0.296	0.128
SDW	0.266	0.259
SLA	0.496	0.159
HL	0.850	0.847
HDW	0.211	0.324
RL	0.209	0.085
RV	–0.002	0.292
RD	0.071	–0.107
RL/SDW	0.095	0.378
RDW	0.073	0.251
R/S	0.310	0.352
S/SR	0.397	0.476
LWC	0.105	0.088

## Discussion

The current study was carried out in a greenhouse using pots filled with sand. This type of pot experiment is a reasonable compromise to avoid the difficulty of phenotyping roots in natural field environments and the unnatural conditions present in hydroponics, aeroponics, or agar plates (Tuberosa, 2012). However, aspects of root growth in this pot system would have still been substantially different from field conditions.

Upon screening the RIL population, this work found significant G×E between control and drought environments for stomatal conductance, leaf number, root length, and root diameter. This G×E was reflected in Q×E detected using the MEA approach for these traits, except for leaf number. MEA is more powerful than the traditional single environment analysis in detecting more significant QTL with higher explained variance. An additional advantage is that it allows quantification of Q×E, because it accounts for G×E and tests all detected QTL in all environments and thus shows their effects in each environment (Crossa and Federer, 2012). Q×E occurs if the QTL effects are strongly expressed in one environment but weakly in another, or if the QTL has opposite effects on the same trait in two different environments (Mackay, 2001; MacMillan *et al.*, 2006; Zhang *et al.*, 2010). Examples of the first case are *LFW1*, *LA1*, *LDW1*, *3,4DW1*, *RL1*, *RL2*, *RV1*, *R/S2*, *HDW3*, and *SDW1*, while examples of the latter case are found for *LA4*, *SLA1*, *RD3*, and *S/SR2*. The latter kind of Q×E obstructs the transferability of QTL mapping results from one environment to another (Mackay, 2001), as selection will be in opposite directions in the two environments.

Knowing about the QTL with opposite effects on several traits in different environments, also known as antagonistic pleiotropy, is of great importance in breeding programmes because breeding for one trait might negatively affect other traits (Rose, 1982; Juenger, 2013). The QTL cluster mapped at the bottom of A7 included a stomatal conductance QTL (*STC1*), which showed signs of antagonistic pleiotropy, with the R-o-18 allele increasing stomatal conductance under control conditions and decreasing it under drought conditions, while having positive effects on biomass under both environments through the colocated *LDW3*, *SDW2*, and *3,4DW3* QTL. However, the similar effect on biomass and the contrasting effect on stomatal conductance could also mean these traits are not allelic, but the result of close linkage of two loci. Further analysis should reveal this.

Stomatal conductance showed clear plasticity, decreasing significantly in the drought environment. Such response is generally correlated with reduced photosynthesis but also with reduced water loss as an adaptive response to drought (Chaves *et al.*, 2003; Condon *et al.*, 2004; Tardieu, 2013). Due to the colocation or antagonistic pleiotropy of the shoot biomass QTL with *STC1*, when comparing both environments, stomatal conductance was negatively correlated with shoot biomass (Table 2B). This reflects an interesting fitness advantage for plants carrying the R-o-18 allele at this QTL cluster, meaning that under drought conditions they show relatively reduced stomatal conductance (contributing to increased drought tolerance) accompanied with relatively increased shoot biomass, compared to plants carrying the L58 allele.

Recently, the plasticity and the evolution of flowering time and water use efficiency (WUE) has been investigated in *B. rapa* under drought environments (Franks, 2011), and the relationship between circadian rhythm, vegetative, and reproductive traits, and leaf gas exchange with the variation of WUE in different watering regimes has been investigated (Edwards *et al.*, 2012). The negative correlation that this work found for stomatal conductance and shoot biomass under drought was also observed by (Edwards *et al.*, 2012), although this was not significant in their study. It also agrees with the positive correlation between WUE and biomass in the drought environment found by these authors and the colocation of WUE and stomatal conductance QTL mapped in *B. rapa* grown under warm and long-day conditions (Edwards *et al.*, 2011). Although the preferred targets for crop improvement in marker-assisted breeding are generally constitutively expressed QTL (Bernardo, 2008), this QTL cluster is attractive to select for, even if it is not constitutive in view of the Q×E observed for *STC1*, because the allele from R-o-18 contributes to increased drought tolerance without having fitness costs due to reducing biomass.

The leaf area response and the underlying QTL in both environments were confirmed by the positive correlation observed between the full RIL screening and QTL reproducibility experiments. Stomatal closure and limited expansion of young leaves under drought have an indirect negative effect on root growth (Chaves *et al.*, 2003; Roycewicz and Malamy, 2012). This was observed by the reduction in root length, concomitant with an increase in root diameter in the drought treatment, corresponding to similar observations reported before for *Brassica* and other crops (Zhu *et al.*, 2011; Edwards *et al.*, 2012; Poorter *et al.*, 2012). It thus appears that, under drought stress in pots, *B. rapa* does not invest in longer roots to take up more water, but in thicker roots to act as a water storage buffer.

Under drought, the R/S ratio increased compared to the well-watered conditions. Biomass allocation under limiting environments can be explained by a functional biomass equilibrium when plants allocate more biomass to roots when the factor limiting growth is below ground (e.g. water or nutrient shortage), to enhance the uptake of that limiting factor (Poorter *et al.*, 2012). The correlation of the R/S ratio with drought tolerance has previously also been documented for *Arabidopsis* and tobacco (Werner *et al.*, 2010), as well as *B. rapa* (Kage *et al.*, 2004; Edwards *et al.*, 2012).

Of the traits examined, G×E was found for most of them, either in the full RIL screening or the reproducibility experiment (Table 1). With so many traits for which G×E was found, it is not surprising that QTL with Q×E were also found, indicating plasticity for many traits. This work used two ways to detect QTL related to phenotypic plasticity, first using the MEA approach (Table 3) and subsequently using the difference between average values per line when comparing both treatments per lines (Table 4). As previously found by Tétard-Jones *et al.* (2011), there is considerable overlap between both methods, but the latter method also detects some novel QTL not found previously. This is probably due to the additional statistical power that can be gained by directly using the phenotypic difference values for mapping, meaning that QTL that did not exceed the threshold in the MEA approach will be detected.

Although almost all traits showed a positive correlation between the results from the full RIL screening experiment and the reproducibility experiment, confirming the initial results, this was not the case for leaf fresh weight and root diameter, suggesting a high level of G×E for those traits, or for water content, where a high environmental effect probably prevented mapping a QTL for this trait. The only trait for which no plasticity QTL was found was chlorophyll content (CHL), which was in line with the inability to detect G×E for this trait (Table 1). However, there was genetic variation for CHL, with four detected QTL (Table 3). There is also a difference in CHL between drought and control conditions, which agrees with previous observations for four *Brassica* species (Ashraf and Mehmood, 1990), but the genotypes appeared to respond similarly to drought exposure by decreasing CHL, explaining the lack of G×E. The four *CHL* QTL mapped to regions previously identified to contain QTL for chlorophyll a and b content in *B. rapa* (Ge *et al.*, 2012), with *CHL1* colocating with one of the three QTL previously identified for chlorophyll fluorescence (Edwards *et al.*, 2011).

Increasing crop productivity under drought conditions is the ultimate goal for marker-assisted breeding programmes. In that respect, the significant antagonistic effect of relatively reduced stomatal conductance along with relatively higher shoot biomass under drought conditions due to the *STC1*/shoot biomass locus at the bottom of chromosome A7 is very interesting, as it suggests that selection on reduced water loss during drought, through reduced stomatal transpiration, is expected to have disproportionately little effect on shoot biomass reduction, which is a favourable combination. In addition, this work reported many QTL underlying several morphological and physiological traits, which appeared to be robust and thus provided the first step towards identifying genes governing those traits. The availability of the whole *B. rapa* genome sequence (Wang *et al.*, 2011) together with possible comparative alignment with the related model species *A. thaliana* (Schranz *et al.*, 2006) will facilitate fine mapping and cloning of candidate genes underlying the desired QTL. This approach will not only be useful in breeding *B. rapa*, but also in breeding other closely related species like *B. juncea* and *B. napus* (Cheng *et al.*, 2011; Li *et al.*, 2013).

## Supplementary material

Supplementary data are available at *JXB* online.

Supplementary Table S1. Phenotypic data of the selected lines for the analysed traits by full RIL screening and QTL reproducibility under control and drought conditions.

Supplementary Data
