# Supplementary material for: Genotype–environment interactions affecting preflowering physiological and morphological traits of Brassica rapa grown in two watering regimes
Source: J Exp Bot. 2014 Jan 27;65(2):697–708. doi: 10.1093/jxb/ert434 (PMC3904722; doi:10.1093/jxb/ert434)
Supplement: Supplementary Data [file supp_65_2_697__index.html]

Genotype–environment interactions affecting preflowering physiological and morphological traits of Brassica rapa grown in two watering regimes — Supplementary Data 

# Genotype–environment interactions affecting preflowering physiological and morphological traits of *Brassica rapa* grown in two watering regimes

## Supplementary Data

Data files

**Files in this Data Supplement:**

- Supplementary Data - Supplementary Data
